# Predictability effects during reading comprehension are not modulated by a visuospatial working memory load

**DOI:** 10.3758/s13421-026-01857-7

**Published:** 2026-01-29

**Authors:** Aine Ito

**Affiliations:** Department of English, Linguistics and Theatre Studies, National University of Singapore, Block AS5, 7 Arts Link, Singapore, 117570 Singapore

**Keywords:** Prediction, Reading comprehension, Working memory, Eye-tracking, Self-paced reading

## Abstract

Predictable words are processed more quickly than unpredictable words during reading, but how this predictability effect is modulated by working memory (WM) limitations is unclear. Two preregistered experiments tested whether the availability of visuospatial WM resources affects reading measures that are sensitive to predictability (gaze duration and skipping probability in Experiment 1, and self-paced reading time in Experiment 2) in native English speakers. The analyses of all measures revealed main effects of predictability and WM load. Their interaction was significant in Experiment 2 but not in Experiment 1. A follow-up analysis on relative reading times in Experiment 2 revealed main effects of predictability and WM load but no interaction between the two, replicating Experiment 1. The apparent interaction in Experiment 2 was likely due to the WM load speeding the overall reading times and masking the predictability effect. Taken together, these findings demonstrate a robust effect of predictability under a cognitively demanding task and suggest that visuospatial WM resources are not necessary for computing probabilities of upcoming words during reading.

## Introduction

Prediction is believed to play an important role in facilitating language comprehension (Kuperberg & Jaeger, 2016; Pickering & Gambi, 2018). For example, predictable words are read more quickly and are more likely to be skipped than less predictable words (for reviews, see Staub, 2015; Wong et al., 2024). However, the robustness and ubiquity of predictability effects are still debated (Heilbron et al., 2022; Huettig & Mani, 2016; Ryskin & Nieuwland, 2023; Wong et al., 2024; Zirnstein et al., 2018). Some studies have addressed this issue by investigating the circumstances under which prediction occurs, and have found that prediction is less likely to occur when working memory (WM) resources are limited (Allison et al., 2025; Ito et al., 2018; Liu et al., 2025). However, which WM components contribute to predictability effects remains unclear. The current study examines the involvement of visuospatial WM, which may support the generation of predictions via event simulation, by activating visual representations of events (Huettig, 2015). To assess the role of visuospatial WM in prediction based on a sentence context, I examined whether a visuospatial WM load interferes with predictability effects during reading.

### Prediction and the role of working memory (WM)

Predictability of a word given a context is one of the factors that predict reading times. According to some theories, cognitive cost for processing each word during sentence comprehension is determined by the surprisal of a word (Hale, 2001; Levy, 2008; though surprisal may not be the only predictor, Roland et al., 2012). Recent large-scale work (Shain et al., 2024) has demonstrated that predictability effects on reading times are logarithmic. This finding was taken as evidence that predictability effects during reading reflect predictive inferences about the likelihood of different interpretations of the sentence, and the processing cost for a word reflects the extent to which this likelihood needs to be updated given that word.

Kuperberg and Jaeger (2016) have also proposed probabilistic predictive inference as a mechanism for prediction. They claim that the degree of prediction depends on the utility of prediction (whether making predictions is advantageous) as the goal of comprehenders is to maximise the utility of prediction (see also Kaan & Grüter, 2021). Thus, prediction is less likely to occur when the disadvantages of prediction outweigh the advantages of prediction compared to when the advantages of prediction outweigh the disadvantages of prediction. This prediction is supported by studies showing that prediction is modulated by factors such as reliability of contextual information and task instructions (Brothers et al., 2017; Chen & Ito, n.d.; H. Kim et al., 2024; Ness & Meltzer-Asscher, 2018, 2021; Zhao et al., 2023).

Another factor that can modulate prediction is WM. WM resources may be required for prediction as contextual information that is used to compute probabilities of upcoming words needs to be temporarily stored in WM. Some recent theories hold that processing cost is determined by the surprisal of a word given a lossy memory representation of the context, rather than complete representations of the context (Futrell et al., 2020; Hahn et al., 2021, 2022). These theories predict that predictability facilitates reading times to a lesser extent when WM is taxed compared to when it is not, because taxing WM should interfere with the construction of contextual representations. However, it is unclear which components of WM are required for prediction. If the role of WM in language prediction is only to temporarily activate linguistic information for generating predictions and to keep it available for prediction, only verbal WM may be required for prediction. However, if other components of WM play an additional role, prediction may be modulated by non-verbal WM.

For example, Huettig (2015) has proposed that one of the mechanisms that accounts for language prediction is event simulation (see also Kuperberg, 2016). According to this account, comprehenders simulate events described in a sentence, activating sensory representations (e.g., visual representations of an event) during language processing (Barsalou, 2009; Grisoni & Pulvermüller, 2022). If the activated visual representations are temporarily stored in WM and used for generating predictions (Altmann & Mirković, 2009; Knoeferle & Crocker, 2007), the availability of visuospatial WM resources may affect predictability effects during reading. Additionally, there is evidence that listeners pre-activate the shape of highly predictable words (Rommers et al., 2013). Taxing visuospatial WM may interfere with pre-activation of visual information, which may subsequently reduce predictability effects.

Alternatively, a domain-general component of WM may contribute to prediction. For example, the central executive includes functions such as focusing and dividing attention, and switching between tasks (Baddeley, 2012). These cognitive capacities may be important for prediction, as prediction may be reduced or fail to occur if the information that cues prediction is not in the comprehender’s attentional focus. Curcic et al. (2019) found that learners of a miniature language based on Fijian used determiner-noun gender-agreement rules to predict the gender of nouns, but this effect was found only among learners who were aware that determiners were useful for prediction. If the ability to focus on useful cues modulates prediction, the ability to perform a concurrent visuospatial WM task (which also involves focusing attention and task switching) may be correlated with the ability to predict language.

Here, I review studies that tested the effects of WM on predictability. These studies suggest that visuospatial WM is required for generating predictions based on linguistic and visual contexts in the visual-world paradigm, where linguistic and visual information is concurrently processed (Huettig et al., 2011). However, the role of visuospatial WM appears more limited than the role of verbal WM, and it is unclear whether visuospatial WM is required for generating context-based predictions during reading. The current study used eye-tracking reading and self-paced reading paradigms because they allow testing prediction without a concurrent visual scene, but the review below focuses on visual-world eye-tracking and event-related potential (ERP) studies, as the most relevant evidence comes from these studies.

### Evidence from the visual-world paradigm

Huettig and Janse (2016) investigated the link between people’s WM capacity (measured using verbal and visuospatial WM tasks) and predictive eye movements in the visual-world paradigm. They found that the degree of L1 Dutch speakers’ prediction based on grammatical gender (e.g., predicting a common-gender object to follow the Dutch article *de*_-common_) was positively correlated with their verbal and visuospatial WM capacity, suggesting the role of WM in making predictive eye movements. Ito et al. (2018) also used the visual-world paradigm and found that prediction based on verb meaning (e.g., predicting a foldable object to follow the verb *fold*) was hindered by an additional verbal WM load of memorising five unrelated words in both L1 and L2 English speakers.

Later studies tested effects of a visuospatial WM load on prediction based on verb meaning in L1 (Wang et al., 2025) and L2 English speakers (Allison et al., 2025). These studies found reduced predictive eye movements with an increased visuospatial WM load, but the visuospatial WM load effects appear smaller than the verbal WM load effects reported in Ito et al. (2018). Liu et al. (2025) tested effects of verbal and visuospatial WM loads on prediction based on Chinese tone sandhi and classifier constraints. They found that both types of load interfered with predictive eye movements, but the verbal WM load did so to a greater extent than the visuospatial WM load, suggesting that verbal and visuospatial WM may play different roles in prediction. These findings suggest that making predictive eye movements requires both verbal and visuospatial WM resources, but it is also possible that the interference occurred in different domains (e.g., the verbal WM load interfered with language processing, and the visuospatial WM load interfered with object encoding).

Other studies found no or weak evidence that predictive eye movements are correlated with people’s WM capacity. Kukona et al. (2016) tested whether meaning-based prediction (e.g., predicting a white cake upon hearing *eat the white*) was predicted by L1 English speakers’ verbal WM capacity (measured using the reading span task; Daneman & Carpenter, 1983) and visuospatial WM capacity (measured using the Corsi block task; Corsi, 1972) and found no evidence for the role of WM. They found that participants’ language skills (e.g., rapid automatised naming score, comprehension skills) were better predictors of their prediction ability. Similarly, Favier et al. (2021) tested syntactic prediction (based on a passive construction) in L1 Dutch speakers and found that participants’ literacy skills (measured using six different tasks) were the strongest predictor of syntactic prediction, and there was no evidence that the verbal WM contributed to syntactic prediction. Additionally, Hintz et al. (2024) conducted a large-scale study involving 487 participants and only found a weak effect of WM (measured using forward and backward digit span tasks) on verb meaning-based prediction in L1 Hungarian speakers. Finally, Perdomo and Kaan (2021) found no evidence that L1 and L2 speakers’ prediction based on contrastive pitch accent in English was modulated by their WM (measured using a forward and backward digit span task in English).

The visual-world studies discussed above investigated prediction based on linguistic and visual information (e.g., which of the depicted objects meets the semantic restrictions of the preceding verb). The exception is Li and Qu (2023), who tested whether prediction based on a highly predictive (i.e., high-cloze) context was related to the verbal WM capacity (measured using the reading span task). They found earlier semantic prediction in the high (vs. low) WM group but similarly early phonological prediction in both groups. The magnitude of these prediction effects did not differ between the two groups, suggesting that the role of WM in context-based prediction may be limited. While the visual-world paradigm is suitable for understanding how people integrate linguistic and visual information, it is difficult to test prediction based on linguistic contexts alone because effects from the visual context need to be considered (Huettig et al., 2011). Additionally, because the visual-world task should involve visuospatial WM (e.g., for temporarily storing object locations), it is unclear whether visuospatial WM resources are required for generating predictions based on a linguistic context.

### Evidence from reading studies

While effects of WM on predictability effects on eye-tracking reading measures are unclear (Wong et al., 2024), some ERP studies examined a relationship between participants’ WM capacity and context-based prediction. Otten and Van Berkum (2009) found that L1 Dutch participants showed an early negativity (a more negative ERP) to articles that were inconsistent in gender with a noun that was predictable from a context (e.g., *de*_-common_ when a neuter noun was predictable) compared to prediction-consistent articles (e.g., *het*_-neuter_). Crucially, this effect did not differ as a function of their verbal WM capacity (measured using the reading span task). However, the lower WM group showed an additional later negativity that was absent in the higher WM group. Otten and Van Berkum interpreted these findings as suggesting that WM affects the way in which people process prediction-disconfirming information rather than the ability to generate predictions.

Ness and Meltzer-Asscher (2018) tested effects of WM on pre-updating (updating of the sentence representations in the WM to include the predicted content). In their study, L1 Hebrew participants read strongly and weakly predictive sentences. ERPs on the verb that preceded the predictable noun were analysed as a measure of pre-updating. The verb elicited a larger P600 effect in the strongly predictive context than in the weakly predictive context. This P600 effect was larger in participants with a higher WM span than those with a lower WM span (measured using a Hebrew version of the reading span task), suggesting that a higher WM span is associated with greater predictive processing.

Similarly, an ERP study by Ding et al. (2023) used Chinese sentences where the context was strongly, moderately or weakly predictive towards a target noun, and the verb that preceded the target boosted the predictability of the target. ERP responses at the verb were used as a measure of prediction generation. The higher WM span group (measured using a Chinese version of the reading span task) showed a larger long-lasting positivity (a more positive ERP) for verbs in the strongly predictive context than in the moderately or weakly predictive contexts. The lower WM span group showed no effect of predictability at the verb. The interaction of predictability by the WM group was taken as evidence that WM modulates prediction generation.

Alemán Bañón and Martin (2024) tested a relationship between WM and lexicosemantic prediction based on a relatedness proportion paradigm from Lau et al. (2013). L1 English and advanced L2 English speakers read semantically related or unrelated prime-target pairs. Both groups showed a smaller N400 for semantically related versus unrelated targets. This N400 effect was larger in a block that contained more related pairs (the proportion of related pairs in the block = 50%) than another block that contained fewer related pairs (10%), suggesting that participants actively predicted (some features of) the targets when the reliability was higher. This N400 effect was not modulated by participants’ WM capacity (measured using the backwards digit span task and the reading span task administered in their L1). However, the N400 effect was found to have low within-subject consistency, which may have made it difficult to reliably test its relationship with WM (Cunnings & Fujita, 2021; Staub, 2021).

In sum, the reviewed studies provide some evidence that WM plays a role in linguistic prediction. Some visual-world studies found effects of visuospatial WM on prediction, but it is difficult to draw conclusions about the effects of visuospatial WM on context-based prediction in the visual-world paradigm, because predictions in the visual world rely on both visual and linguistic information. Some ERP studies examined the relationship between WM and predictability effects during reading, but the role of visuospatial WM remains unclear in these studies as they did not include a visuospatial WM task. Therefore, it is unclear whether context-based prediction during reading requires visuospatial WM.

### Current study

To understand the role of visuospatial WM in prediction during reading, the current study tested whether context-based prediction is hindered by a secondary visuospatial WM task (the Corsi block-tapping task; Corsi, 1972). The Corsi block-tapping task involves the encoding and maintenance of visuospatial information and is widely used for measuring visuospatial WM (see Fischer, 2001, for a review). Following prior studies (Allison et al., 2025; Ito et al., 2018; Liu et al., 2025), the WM load was manipulated within subjects to test a causal relationship between WM and prediction. Multiple levels of load were not created to maximise the number of observations in each condition.

Context-based prediction was measured by comparing reading measures that should be sensitive to predictability (gaze duration and skipping probability in Experiment 1 and self-paced reading times in Experiment 2) in predictable and unpredictable conditions. Eye-tracking reading and self-paced reading paradigms were used, as these methods allow testing context-based predictability effects on online reading measures and have been widely used to test predictability effects (Amenta et al., 2023; Andrews et al., 2023; Bondoc & Schafer, 2024; Brothers & Kuperberg, 2021; Cutter et al., 2023; Smith & Levy, 2013; Staub, 2015; Wong et al., 2024). However, these methods have important differences which can affect the effect of interest (e.g., skipping and re-reading are disallowed in self-paced reading), which are discussed in the *General discussion* section.

If visuospatial WM resources are required for context-based prediction, predictability effects should be smaller when participants read sentences with (vs. without) the additional WM task in both experiments. Although facilitation for predictable versus unpredictable words could be driven by easier integration of the predictable words into the ongoing contexts, it is unlikely that predictability effects are solely driven by late integration processes because predictability affects very early eye-movement measures (e.g., skipping), and predictability does not specifically affect long fixations that likely reflect integration difficulty (Staub, 2015). However, as lexical integration can affect early measures via parafoveal processing (Veldre et al., 2020), predictability effects during reading may reflect both prediction and integration processes (Wong et al., 2024). Self-paced reading measures are also likely to reflect both prediction and integration processes, as it is not straightforward to dissociate the two processes. I return to these points in the *General discussion* section. Both experiments were preregistered (Experiment 1: https://osf.io/ktypr, Experiment 2: https://osf.io/jx48b). The study was approved by the departmental ethics review committee at the National University of Singapore.

## Experiment 1

### Methods

#### Participants

The final sample included 81 L1 English speakers (mean age = 22, range = 18–31 years; 26 males, 55 females). The sample size was determined based on Brysbaert and Stevens’ (2018) recommendation that a properly powered experiment has at least 1,600 observations per condition to detect a two-way interaction. This experiment comprised 88 items and four conditions (yielding 22 observations per condition per participant), and these items were presented in eight experimental lists. To achieve 1,600 observations per condition and keep the participant count divisible by eight, 80 was chosen as the target sample size (preregistration: https://osf.io/ktypr).

Two additional participants were excluded because more than 20% of their data were lost due to track loss or blinks. No participant was excluded for low comprehension question accuracy. All participants reported they spoke other language(s). Additional languages participants spoke are summarised in the Online Supplementary Material on the Open Science Framework (OSF) at https://osf.io/7zvhg/.

#### Stimuli

Eighty-eight items were selected from a candidate set of 171 items adapted from Brothers and Kuperberg (2021) and Frisson et al. (2017). The target word (e.g., *air*) was highly predictable from the context in the high-cloze condition (e.g., *In polluted cities, you can barely breathe the air outside the building*) and unpredictable in the low-cloze condition (e.g., *They suspected there was something wrong with the air outside the building*). The target word was never sentence-final and was followed by the same continuation in both conditions. The mean length of the target words was five letters (range = 3–8).

The predictability was assessed in a cloze test, in which 20 L1 English speakers who did not participate in the eye-tracking experiment read a sentence fragment up to the target word and completed the fragment with the first continuation that came to mind. Any spelling errors were corrected, and singular and plural completions were scored as the same word. The cloze probability was computed in two ways: (a) by scoring completions that started with the target word (e.g., *air*), and (b) by scoring completions that contained the target word anywhere in the response (e.g., *fresh air*). The mean cloze probability for the predictable target was 94.5% when only target-word-initial completions were scored and 95.2% when completions that contained the target word were scored (both cloze values ranged from 85% to 100%). The cloze probability for the unpredictable target word was 0. A yes-no comprehension question was created for each item, which was identical within each item (e.g., *Was the air quality considered poor?*).

#### Procedure

**Fig. 1:**
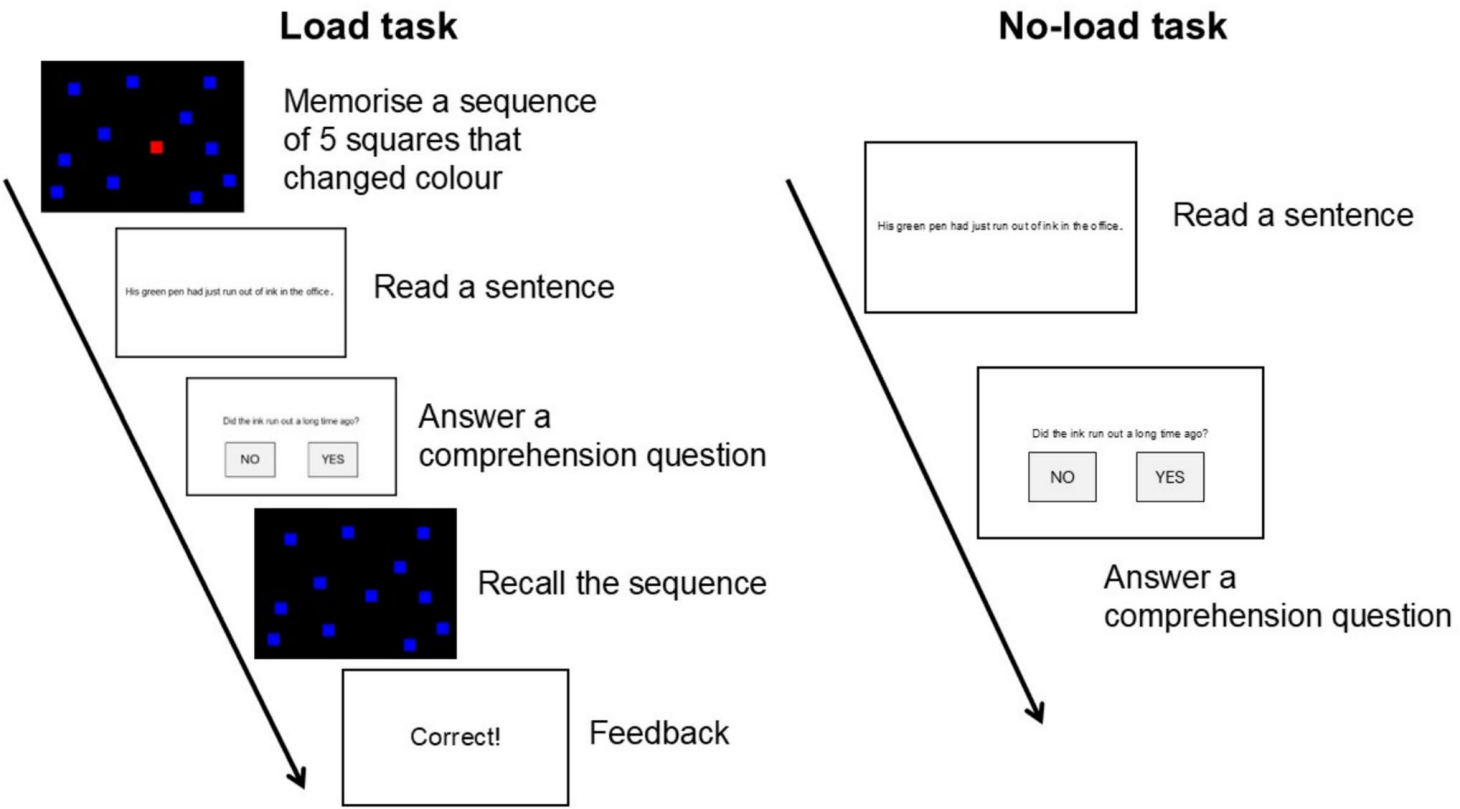
An illustration of the load and no-load task

Participants’ eye movements were recorded using an EyeLink 1000 Plus Desktop mount eye tracker sampling at 1,000 Hz. Interest areas were created using the auto-segmentation function in SR Research Experiment Builder. The eye tracker was calibrated using the nine-point calibration grid at the beginning of the experiment and whenever necessary. The sentences were presented on a viewing monitor at a resolution of 1,024 × 768 pixels. In the no-load task, participants were asked to read sentences silently and answer a yes-no comprehension question about the sentence they just read. In the load task, participants were additionally asked to perform a concurrent memory task (Fig. 1). The task was blocked, and the order of the task was counterbalanced such that half of the participants received the load task first. Participants were presented with each item once and completed an equal number of trials in each condition and task. The stimuli in the experiment were pseudo-randomised within each task so that there were no more than three consecutive trials in the same condition.

Each trial started with a drift check. In the no-load task, participants fixated a cross presented just to the left of the first character of the sentence. The sentence appeared on a single line when they fixated the cross for at least 100 ms. Sentences were displayed in 10-point Courier New font. Participants clicked the mouse when they finished reading. Then, a comprehension question appeared. Participants answered it by clicking a yes or no button on the screen.

In the load task, participants clicked the mouse to begin the trial after a drift check. They heard a beep and saw 12 blue squares. Five of the squares turned red one by one, and participants were instructed to memorise the sequence. They then read a sentence and answered a comprehension question (the same procedure as in the no-load task). After that, they saw the 12 blue squares again and were instructed to click the five squares that had changed colour in the same order. The number of squares to be recalled – namely, five – was decided based on a pilot to avoid ceiling and floor effects in the recall performance, and it was fixed for all trials. To prevent participants from prioritising the sentence reading task, feedback (correct or wrong) was provided for the recall accuracy on every trial.

#### Data exclusion

Following the preregistration, trials with track loss and trials that contained a blink in the target word region were excluded, but no trials were excluded based on the comprehension question accuracy. Short fixations (< 80 ms) within 1° of visual angle of another preceding or following fixation were automatically merged. Other fixations less than 80 ms or more than 800 ms in duration were excluded. In addition to the preregistered data cleaning, automatic drift correction was performed in SR Research Data Viewer because some fixations were slightly below or above the interest areas. This aligned all fixations during the sentence presentation vertically to the average location along the Y-dimension, except for the fixations that deviated more than 30 pixels in the Y-dimension from the mean Y position. This was performed for trials that contained fixations above or below the critical interest areas. When all fixations were consistently above or below the interest areas, they were manually corrected but only along the Y-dimension. The proportion of excluded trials was 4% in the high-cloze, load condition; 5% in the high-cloze, no-load condition; 4% in the low-cloze, load condition; and 5% in the low-cloze, no-load condition.

#### Data analysis

A linear-mixed effects model tested effects and an interaction of predictability (sum-coded as 1 = high-cloze, ˗1 = low-cloze) and task (sum-coded as 1 = load, ˗1 = no-load) on log-transformed gaze duration for the target word. A generalised linear-mixed effects model tested the same effects and interaction on skipping probability for the target word (coded binomially as 1 = skipped, 0 = not skipped). Both models were first fit with the maximal random-effects structure justified by the design but later simplified by dropping the random effects that accounted for the least variance due to non-convergence or singular fit. The final model for gaze duration included by-participant and by-item random intercepts and by-participant random slopes for predictability and task. The model for skipping probability converged with the maximal random-effects structure. Accuracy of the recall task was computed as the proportion of trials in which all five locations were recalled in the correct order.

### Results: Preregistered analysis

#### Recall and comprehension performance

The mean recall accuracy in the load task was 64% (*SD* = 19, range = 25–95), suggesting no ceiling or floor effect. The mean accuracy for the comprehension questions was 93% (*SD* = 4, range = 76–99) overall, and it was similar in the load (93%, *SD* = 5, range = 77–100) and no-load (93%, *SD* = 4, range = 75–100) tasks.

#### Gaze duration

**Fig. 2:**
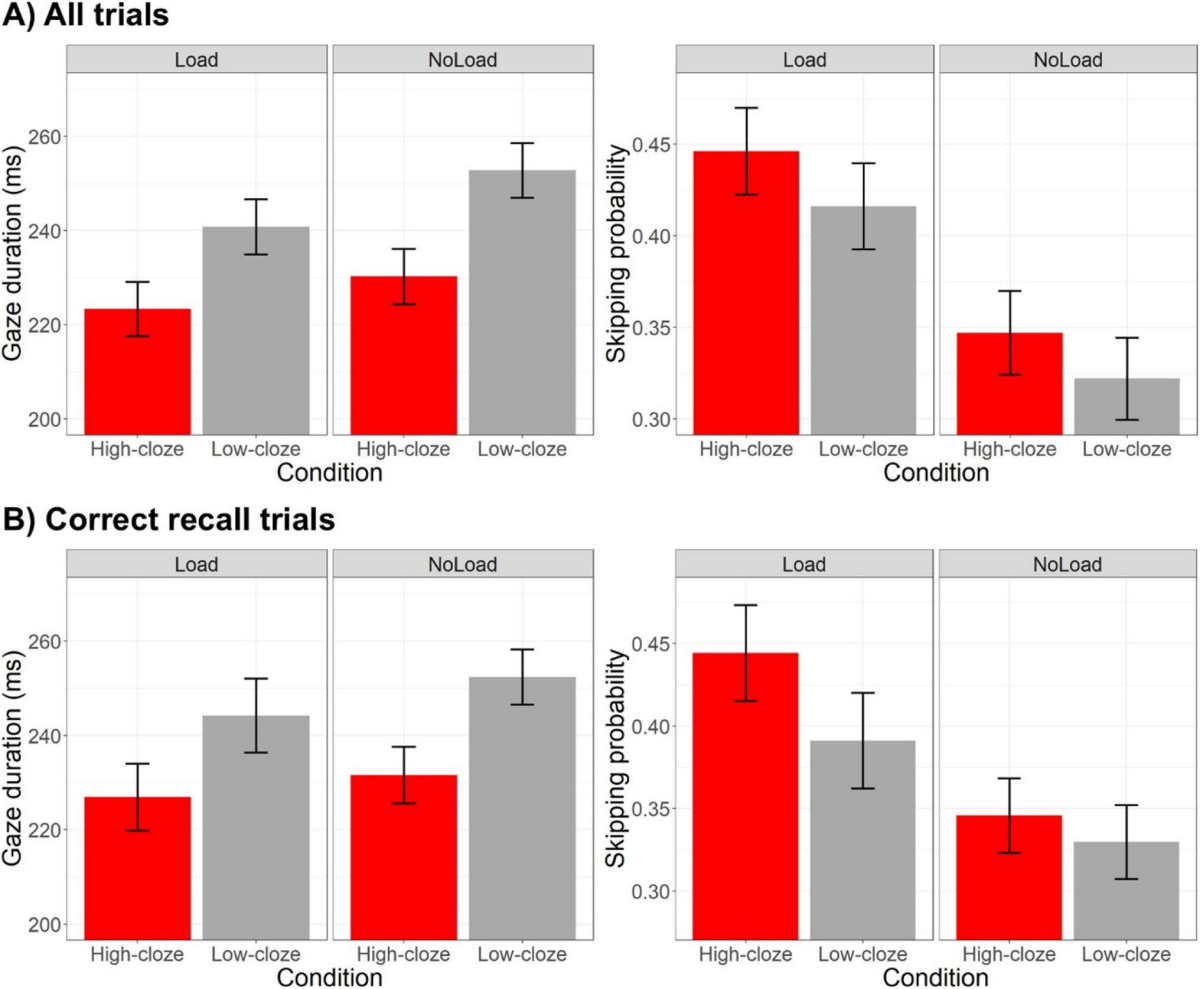
(**A**) Results from all trials, and (**B**) results from correct recall trials in Experiment 1. Gaze duration in milliseconds (left panel) and skipping probability (right panel) for each condition with 95% confidence intervals

The analysis on gaze duration revealed a main effect of predictability, β = ˗0.04, *SE* = 0.005, *t* = ˗7.8, and a main effect of task, β = ˗0.02, *SE* = 0.007, *t* = ˗2.7 (Fig. 2A). Predictability and task did not interact, β = 0.004, *SE* = 0.005, *t* = 0.7. These effects suggest a shorter reading time for predictable versus unpredictable words (228 ms vs. 247 ms) and in the load versus no-load task (233 ms vs. 242 ms), but there was no evidence that the task modulated the predictability effect.

#### Skipping probability

The analysis on skipping probability revealed a main effect of predictability, β = 0.07, *SE* = 0.03, *z* = 2.1, *p* =.04, and a main effect of task, β = 0.22, *SE* = 0.04, *z* = 5.9, *p* <.001 (Fig. 2A). These factors did not interact, β = 0.010, *SE* = 0.03, *z* = 0.29, *p* =.8. These effects indicate a higher skipping probability for predictable versus unpredictable words (39% vs. 37%) and in the load versus no-load task (42% vs. 34%). As in the gaze duration results, there was no evidence that the task modulated the predictability effect.

### Results: Exploratory analysis

#### Predictability by task interaction in correct recall trials

The large variability in the recall performance may suggest that some participants prioritised the reading task (despite the feedback on the recall task). If so, they may be unaffected by the WM load, whereas participants who put equal weight on both tasks may show a predictability by task interaction. To test this, I repeated the analysis above on a subset of data only including trials in which the locations of all five squares were correctly recalled (64% of the load trials). The analysis on gaze duration showed a main effect of predictability, β = ˗0.04, *SE* = 0.007, *t* = ˗6.3, and a marginally significant effect of task, β = ˗0.01, *SE* = 0.007, *t* = ˗1.9, but the interaction was not significant, β = 0.004, *SE* = 0.006, *t* = 0.8 (Fig. 2B). Similarly, the analysis on skipping probability showed a main effect of predictability, β = 0.08, *SE* = 0.03, *z* = 2.7, *p* =.008 and a main effect of task, β = 0.21, *SE* = 0.03, *z* = 6.9, *p* <.001, but the interaction was not significant, β = 0.040, *SE* = 0.03, *z* = 1.3, *p* =.2. These results replicate the preregistered analysis and suggest that the visuospatial WM load did not modulate prediction.

#### Predictability by task by recall performance interaction

The recall performance suggests a large variability in participants’ WM capacity, and it is possible that participants with lower WM capacity are more subject to the additional WM load. To test this possibility, I calculated the mean recall accuracy for each participant and tested a three-way interaction of predictability by WM load by recall accuracy on gaze duration and skipping probability. The three-way interaction was not significant in gaze duration, β = 0.02, *SE* = 0.03, *t* = 0.8, or skipping probability, β = 0.07, *SE* = 0.15, *z* = 0.5, *p* =.6. Thus, there was no evidence that the predictability by load interaction depends on participants’ WM capacity. However, these results need to be interpreted with caution because the sample size in this experiment was estimated based on a 2 × 2 design, and the experiment may not have enough statistical power to test the three-way interaction. The main effect of recall accuracy and other interactions involving recall accuracy were not significant in the gaze duration analysis, |*t*|s < 1.1, or in the skipping probability analysis, *p*s >.16. Full results are available on the Open Science Framework (OSF) repository.

### Discussion

Experiment 1 replicated the predictability effects on gaze duration and skipping probability. It also showed that the visuospatial WM load shortened gaze duration and increased skipping probability. The lack of the predictability by task interaction in both measures is consistent with the hypothesis that context-based prediction does not require visuospatial WM resources. However, consistent evidence from another methodology would reinforce this conclusion. To test the role of visuospatial WM resources in context-based prediction using the same experimental materials, Experiment 2 aimed at replicating Experiment 1 using the web-based self-paced reading paradigm.

## Experiment 2

### Methods

#### Participants

The final sample included 125 L1 English speakers (mean age = 26, range = 21–35 years; 41 males, 83 females, one other), and they were recruited via university mailing lists and internet forums. The sample size was increased from Experiment 1 because data from a web-based experiment were expected to be noisier. All participants reported they spoke other language(s). Additional languages participants spoke are summarised in the Online Supplementary Material at https://osf.io/7zvhg/. Nine additional participants were tested but excluded because more than 30% of the trials were excluded from their data (*N* = 6; this exclusion was not specified in the preregistration, but it was deemed necessary as some of these participants likely kept the space bar pressed to let the sentence advance very quickly) or because they answered more than 30% of the comprehension questions incorrectly (*N* = 3).

#### Stimuli and procedure

The stimuli were identical to Experiment 1. Unlike in Experiment 1, participants completed the experiment on PCIbex Farm (https://farm.pcibex.net/) using their laptop or desktop computer (a tablet was not allowed). They were asked to complete the experiment at a quiet location where they were able to concentrate. The sentences were presented one word at a time using the moving window paradigm. Participants answered yes-no comprehension questions by pressing J for ‘yes’ and F for ‘no’ on the keyboard.

#### Data exclusion

Reading times below 100 ms and above 3,000 ms were excluded first. The lower cut-off value was changed from the preregistered 200 ms because the original threshold resulted in more than 30% data exclusion, which was much higher than other self-paced reading studies (e.g., Haeuser & Kray, 2022; Lyu et al., 2024; they used a 100-ms cut-off). Then, reading times beyond 2 SDs from the mean (calculated for each condition for each participant) were also excluded. As in Experiment 1, no trials were excluded based on the comprehension question accuracy. The proportion of excluded data for each condition was 6% in the high-cloze, load condition; 5% in the high-cloze, no-load condition; 6% in the low-cloze, load condition; and 5% in the low-cloze, no-load condition.

#### Data analysis

A linear-mixed effects model tested main effects and an interaction of predictability (sum-coded as 1 = high-cloze, ˗1 = low-cloze) and task (sum-coded as 1 = load, ˗1 = no-load) on log-transformed reading times for the target word. The final model included by-participant and by-item random intercepts, by-participant random slopes for predictability and task, and a by-item random slope for predictability.

### Results: Preregistered analysis

#### Recall and comprehension performance

The mean recall accuracy in the load task was 54% (*SD* = 24, range = 0–95), suggesting no ceiling or floor effect as in Experiment 1. The mean accuracy for the comprehension questions was 91% (*SD* = 5, range = 77–100) overall, and it was similar in the load (91%, *SD* = 5, range = 77–100) and no-load (91%, *SD* = 5, range = 77–100) tasks.

#### Reading time

**Fig. 3:**
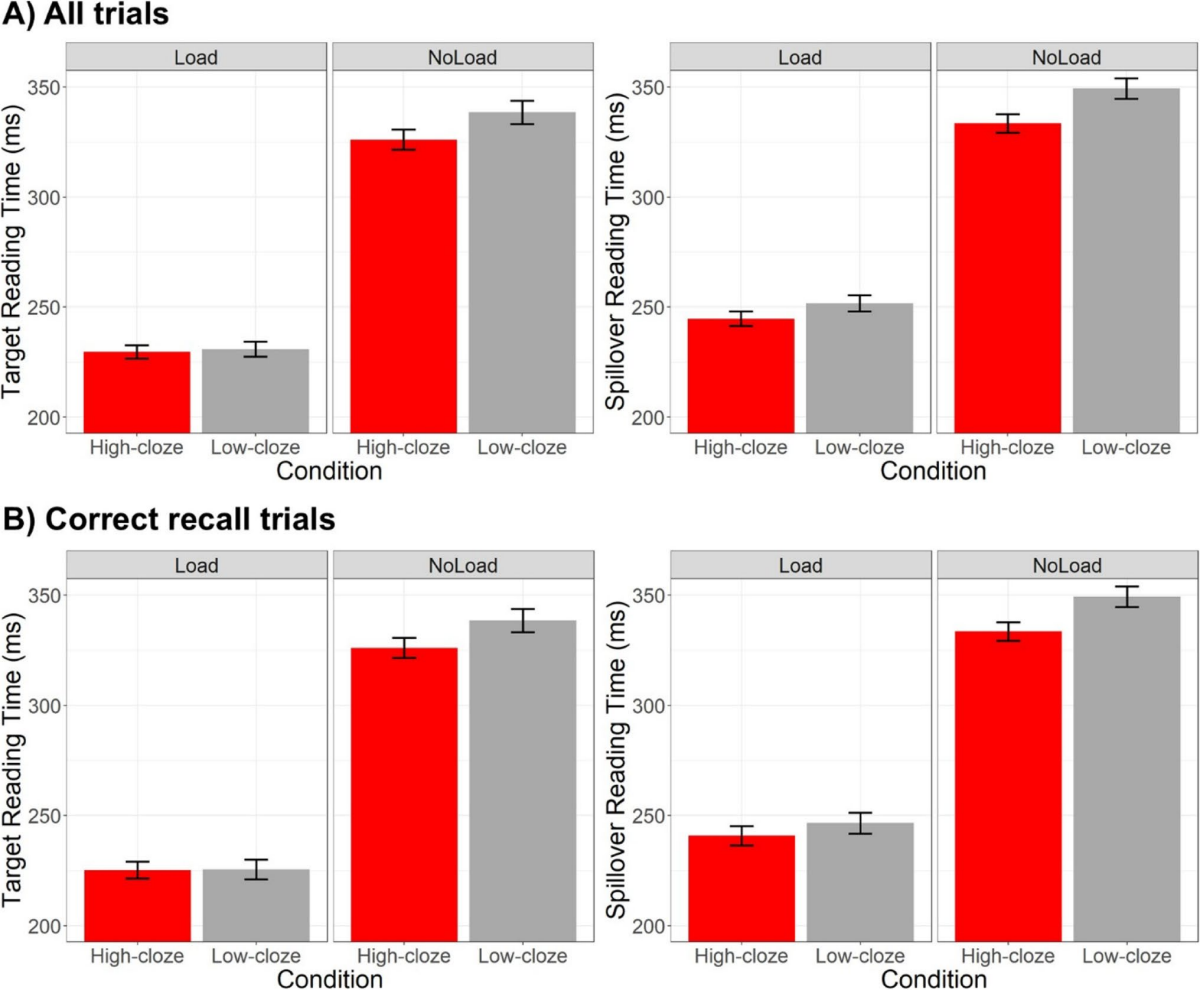
(**A**) Results from all trials, and (**B**) results from correct recall trials in Experiment 2. Self-paced readings times in the target region (left panel) and the spillover region (right panel) for each condition with 95% confidence intervals

The analysis revealed a main effect of predictability, β = ˗0.008, *SE* = 0.003, *t* = ˗3.1, a main effect of task, β = ˗0.18, *SE* = 0.008, *t* = ˗22.1, and a significant interaction of predictability by task, β = 0.007, *SE* = 0.002, *t* = 3.4 (Fig. 3A, left). Follow-up pairwise tests with Tukey’s correction showed a significant effect of predictability in the no-load task, β = ˗0.03, *SE* = 0.007, *z* = ˗4.6, *p* <.001 (high-cloze: 326 ms vs. low-cloze: 339 ms), but not in the load task, β = ˗0.002, *SE* = 0.007, *z* = ˗0.25, *p* =.8 (high-cloze: 230 ms vs. low-cloze: 231 ms).

### Results: Exploratory analysis

#### Predictability by task interaction in correct recall trials

As in Experiment 1, the analysis above was repeated on a subset of data with correct recall trials (54% of the load trials). This analysis showed a main effect of predictability, β = ˗0.007, *SE* = 0.003, *t* = ˗2.3, and a significant effect of task, β = ˗0.18, *SE* = 0.008, *t* = ˗21.5, and a significant interaction of the two, β = 0.008, *SE* = 0.002, *t* = 3.0 (Fig. 3B, left). Follow-up pairwise tests with Tukey’s correction showed a significant effect of predictability in the no-load task, β = ˗0.03, *SE* = 0.007, *z* = ˗4.2, *p* <.001 (high-cloze: 326 ms vs. low-cloze: 339 ms), but not in the load task, β = 0.0002, *SE* = 0.009, *z* = 0.02, *p* =.9 (high-cloze: 225 ms vs. low-cloze: 225 ms). These results replicate the preregistered analysis and suggest that the visuospatial WM load eliminated the predictability effect.

#### Predictability by task by recall performance interaction

**Fig. 4:**
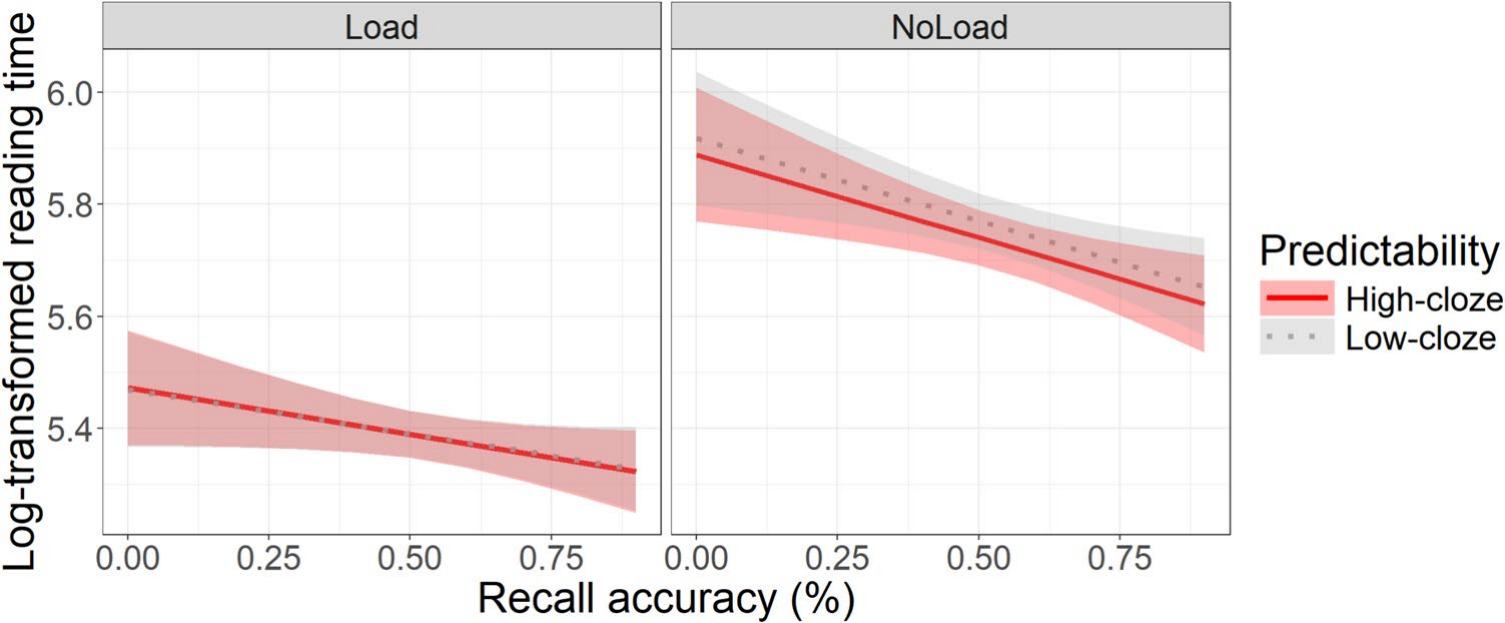
Estimated marginal means from the three-way interaction model in Experiment 2, which found a marginally significant interaction of task and recall accuracy

As in Experiment 1, I tested a three-way interaction of predictability by load by recall accuracy. This analysis showed a significant main effect of recall accuracy, β = ˗0.23, *SE* = 0.088, *t* = ˗2.6, and a marginally significant two-way interaction of task by recall accuracy, β = 0.067, *SE* = 0.034, *t* = 2.0, but the three-way interaction was not significant, β = ˗0.002, *SE* = 0.008, *t* = ˗0.2 (Fig. 4). Thus, participants with a higher recall accuracy showed faster reading times, and this effect was slightly stronger in the no-load task, but the predictability effect was unaffected by the recall performance.

#### Spillover region

Reading times in the spillover region (the next word) were also analysed as predictability effects may be larger in this region (Brothers & Kuperberg, 2021; Smith & Levy, 2013). This analysis showed a main effect of predictability, β = ˗0.016, *SE* = 0.002, *t* = ˗7.6, and a significant effect of task, β = ˗0.16, *SE* = 0.008, *t* = ˗21.7, and a significant interaction of the two, β = 0.005, *SE* = 0.002, *t* = 2.2 (Fig. 3A, right). Follow-up pairwise tests with Tukey’s correction showed a significant but smaller effect of predictability in the load task, β = ˗0.023, *SE* = 0.006, *z* = ˗3.7, *p* = <.001 (high-cloze: 245 ms vs. low-cloze: 252 ms) than in the no-load task, β = ˗0.042, *SE* = 0.006, *z* = ˗6.9, *p* <.001 (high-cloze: 334 ms vs. low-cloze: 349 ms). Thus, the predictability effect emerged on the next word in the load task, and this effect was smaller than in the no-load task, but it was significant in both tasks. The analysis only including correct recall trials also showed main effects of predictability, β = ˗0.017, *SE* = 0.003, *t* = ˗5.7, and task, β = ˗0.16, *SE* = 0.008, *t* = ˗20.6, but the interaction of the two was not significant, β = 0.004, *SE* = 0.003, *t* = 1.5.

#### Relative reading time analysis

**Fig. 5:**
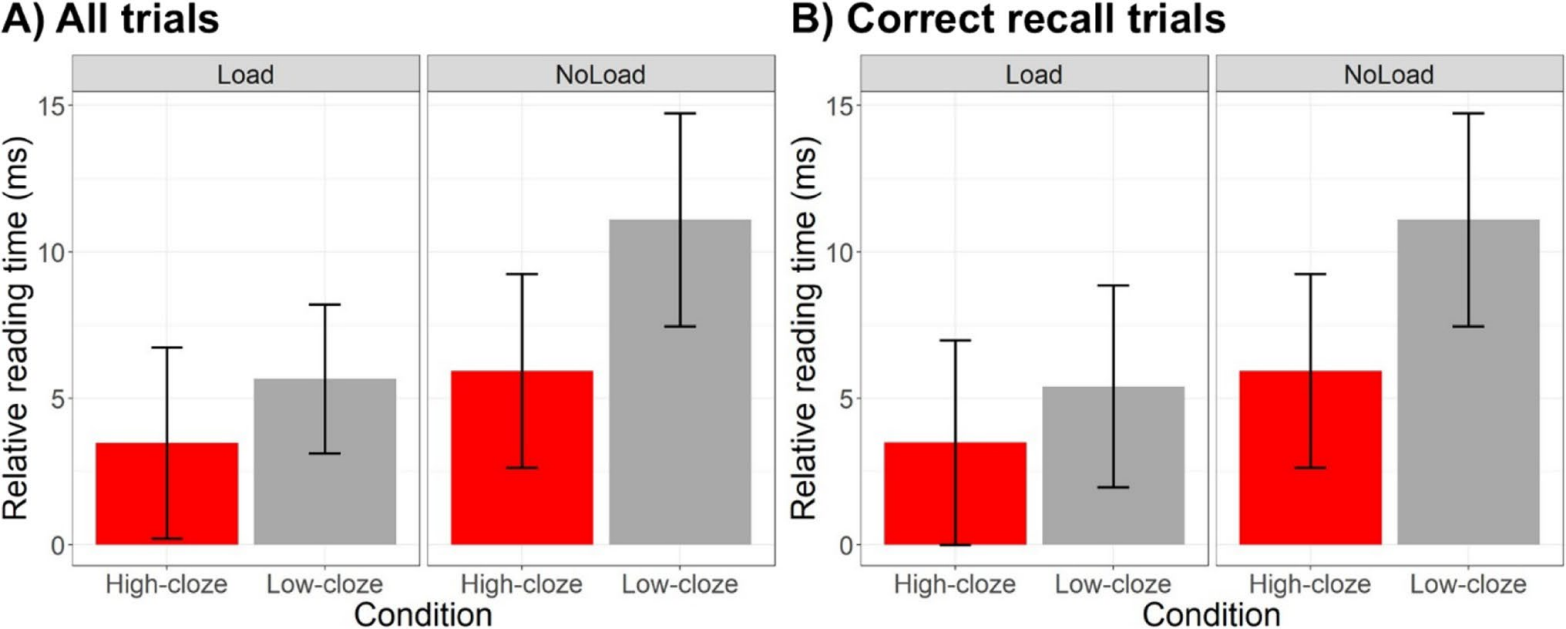
(**A**) Relative reading times from all trials, and (**B**) relative reading times from correct recall trials in Experiment 2. Mean relative reading times in milliseconds for each condition with 95% confidence intervals

The preregistered analysis on reading times showed a robust main effect of load, which was more pronounced than in Experiment 1. The very fast self-paced reading times in the load task may have masked the predictability effect that would have been otherwise present. To explore this possibility, I computed relative reading times by subtracting reading times for the pre-target word from reading times for the target word (which indicates how much faster or slower participants read the target word relative to the previous word). I then tested main effects and an interaction of predictability and task on this measure. The final model for this analysis included by-participant and by-item random intercepts. Relative reading times were not log-transformed because some values were negative. This analysis revealed main effects of predictability, β = ˗1.80, *SE* = 0.81, *t* = ˗2.2 (high-cloze: 5 ms vs. low-cloze: 8 ms), and task, β = ˗1.94, *SE* = 0.81, *t* = ˗2.4 (load: 5 ms vs. no-load: 9 ms). The interaction of the two was not significant, β = 0.75, *SE* = 0.81, *t* = 0.93. The same analysis on trials with correct recall showed no significant main effects or interaction, |*t*|s < 2. Thus, when the overall faster reading times in the load task were taken into account, there was no evidence that the predictability effect was modulated by the visuospatial WM load, and predictability facilitated reading times in both tasks, consistent with Experiment 1 (Fig. 5).

## General discussion

The two experiments replicated previous findings that predictable words are more likely to be skipped and read faster than unpredictable words (Brothers & Kuperberg, 2021; Shain et al., 2024; Staub, 2015; Wong et al., 2024), suggesting that participants engaged in predictive processing during reading. Participants were also more likely to skip the target word and read it faster overall when they were under the visuospatial WM load compared to when they were not. Faster reading under the WM load may reflect participants’ strategy to shorten the time until the recall task. Crucially, the visuospatial WM load did not affect the predictability effects in Experiment 1. The visuospatial WM load eliminated (in the target word region) or weakened (in the spillover region) the predictability effects in Experiment 2, but this interaction seems to have been driven by the robust effect of load masking the predictability effects, as the relative reading time analysis did not show a significant interaction.

## The role of visuospatial WM in prediction

The predictability effects during eye-tracking reading were not modulated by the visuospatial WM load, suggesting that the role of visuospatial WM is minor during natural reading. As discussed earlier, the predictability effects in the current study may reflect both prediction and integration processes, and these processes are difficult to tease apart in reading measures. However, no interaction of predictability by WM load suggests that neither prediction nor integration was affected by the availability of visuospatial WM resources, supporting the conclusion that context-based prediction during reading is largely unaffected by the availability of visuospatial WM resources. Therefore, predictions may have been generated primarily based on non-visual information (e.g., linguistic representations) in the current study.

This result is striking considering the difficulty of the WM task, as the recall accuracy suggests. A similar lack of visuospatial WM effects on language comprehension has been reported in Fedorenko et al. (2007). They also used a dual-task paradigm and found that a verbal WM load modulated effects of syntactic complexity (subject-extracted vs. object-extracted relative clauses) during reading, but a visuospatial WM load did not, suggesting that visuospatial WM resources are not used for syntactic operations. The current study further suggests that generating linguistic predictions does not use visuospatial WM resources either.

Alternatively, visuospatial WM may play a role when the target word is predictable based on a described event or a larger discourse context rather than on contextual meaning or syntactic structure. Prediction may rely on a mechanism involving event simulation (Huettig, 2015; Kuperberg, 2016), but the involvement of this mechanism may be limited to cases in which comprehension involves building connections among multiple events (e.g., when reading a storybook). In the current study, predictability effects were measured by comparing very high-cloze and zero-cloze sentences. The high-cloze contexts often contained words that were highly associated with the target word (e.g., in one of the high-cloze sentences *In polluted cities, you can barely breathe the air outside the building*, the contextual words *polluted* and *breathe* are semantically associated with the target word *air*). In such a context, the meaning of these contextual words may cue prediction of the target word without involving event simulation (Chow et al., 2016; Kukona, 2020; Kukona et al., 2011). However, in other contexts, representations related to a described event may cue prediction. For example, Metusalem et al. (2012) found that event representations of a sentence context can facilitate processing of a word that is associated with the event but not with words in the context (e.g., the event of *playing in the snow* is associated with *jacket*, but *snow* and *jacket* are not semantically associated). Generating predictions using such event representations may engage visuospatial WM. Future research can investigate whether a visuospatial WM load affects prediction generated based on a broader discourse or social context, as such predictions should involve the integration of linguistic and non-linguistic cues and be more likely to involve event simulation (e.g., by combining linguistic information with speaker characteristics or a wider discourse; Brothers et al., 2017; Münster & Knoeferle, 2018; Ness & Meltzer-Asscher, 2021; Nieuwland & Van Berkum, 2006; Van Berkum et al., 2008).

The absence of an interaction between predictability and WM load is inconsistent with the possibility that a domain-general component of WM (e.g., executive functions) contributes to context-based prediction. The concurrent visuospatial WM task used in the current study should engage the capacities to focus attention and to divide attention across different tasks, but prediction was not affected by this dual task. Thus, the contribution of WM may be primarily the storage and maintenance of linguistic information (i.e., the phonological loop; Baddeley, 2000). However, there may be situations where executive functions play a role in prediction, for example, when focusing on a particular cue is key to prediction.

## Inconsistency between the eye-tracking and self-paced reading measures

Experiment 2 (self-paced reading) revealed a larger predictability effect in the no-load than in the load task. However, the analysis on relative reading times suggests that the interaction was likely driven by the very fast reading times in the load task masking the predictability effect. The WM load also shortened gaze duration and increased skipping probability in Experiment 1 (eye tracking), but these effects did not reduce the predictability effects in the load task. Thus, the WM load may have interfered with processes specific to the self-paced reading paradigm. For example, the nature of the self-paced reading paradigm (e.g., word-by-word presentation) may have increased cognitive demands, making its measure more subject to a WM load. After all, readers normally do not press a key to read the next word, and the self-paced reading paradigm disallows natural reading behaviours like skipping and re-reading, so task demands were likely to be higher in the self-paced reading task than in the eye-tracking reading task. Not being able to re-read previous words could have affected the quality of the contextual representations, as comparing the current input with previously encountered words may help to integrate the current input into the ongoing context.

It is also possible that participants in Experiment 2 were more likely to strategically speed up their reading to optimise the recall performance. In the self-paced reading task, readers may use more goal-oriented mechanisms or domain-general mechanisms that also support the visuospatial WM task. In line with this possibility, some studies have found that the domain-general network is engaged during language comprehension when there is an explicit task but not during passive listening or reading (Diachek et al., 2020; Wright et al., 2011). To address these possibilities, it would be interesting to replicate the current study using an ERP with the rapid serial visual presentation (RSVP) paradigm or auditory stimulus presentation to fix the presentation rate. Eye-tracking reading and self-paced reading have been widely used to test similar psycholinguistic questions, but it remains unclear whether reading time measures from these paradigms directly map onto each other. The findings from the current experiments signal the importance of replication using different experimental paradigms.

An alternative explanation for the between-experiment inconsistency is that self-paced reading times may reflect both early and late lexical processes that gaze duration may not be sensitive to (e.g., ease of lexical integration into the context), and the interaction found in Experiment 2 may suggest that the WM load modulates late integration processes instead of early predictive processing. To test this possibility, I conducted an exploratory analysis testing the interaction and main effects of predictability and task on (log-transformed) total reading time in Experiment 1 because total reading time should be sensitive to both early and late lexical processes (Vasishth et al., 2013), and it should resemble self-paced reading time in that sense. This analysis showed significant main effects of predictability, β = ˗0.055, *SE* = 0.009, *t* = ˗6.0, and task, β = ˗0.034, *SE* = 0.010, *t* = ˗3.5, but no interaction of the two, β = 0.004, *SE* = 0.008, *t* = 0.48 (the descriptive statistics and the plot are available in the OSF repository). Thus, there was no evidence that the predictability effect on total reading time was modulated by the WM load, suggesting the robust effect of predictability during natural reading.

The recall performance was slightly lower in Experiment 2 (54%) than in Experiment 1 (64%). If participants in Experiment 2 had lower WM capacity than participants in Experiment 1, this could explain the inconsistency because people with lower WM capacity may be more susceptible to the additional WM load. However, the three-way interaction of predictability by WM load by recall performance was not significant in either experiment, suggesting that the between-experiment inconsistency is unlikely to be due to individual differences in recall performance. An additional analysis excluding participants whose mean recall accuracy was below 30% (five participants in Experiment 1, and 25 participants in Experiment 2) also replicated the results from the preregistered analysis (for details, see E1_script and E2_script on the OSF repository), indicating that the current results were not solely driven by participants with low recall accuracy. Finally, another difference between the experiments was whether the experiment was conducted in the laboratory (Experiment 1) or on the internet (Experiment 2). While Experiment 2 recruited more participants to compensate for the likely noisier data in a web-based experiment, other uncontrolled factors may have contributed to the inconsistency.

## Conclusions

The current eye-tracking and self-paced reading experiments revealed robust effects of predictability during reading, even when participants read sentences under a visuospatial WM load. Although the WM task sped up reading and increased skipping probability, it did not eliminate predictability effects. These results suggest a minimal role of visuospatial WM and executive functions of WM in context-based prediction during natural reading in L1.

## Declarations

### Data Availability

The materials and data for both experiments are available at https://osf.io/7zvhg/.

### Code availability

The analysis scripts for both experiments are available at https://osf.io/7zvhg/.

### Open practices statement

The materials, data and analysis scripts for both experiments are available at https://osf.io/7zvhg/. Both experiments were preregistered (Experiment 1: https://osf.io/ktypr, Experiment 2: https://osf.io/jx48b).
